# From battlefield to living room: a community-based narrative intervention for reciprocal healing during war

**DOI:** 10.3389/fpubh.2026.1776917

**Published:** 2026-02-23

**Authors:** Oren Cohen Zada

**Affiliations:** 1 Ono Academic College, Kiryat Ono, Israel; 2 The Graduate School, Talpiot Academic College of Education, Holon, Israel

**Keywords:** active container, acute stress, community resilience, narrative intervention, reciprocal healing, shared trauma

## Abstract

**Background:**

The “Swords of Iron” war created a reality of shared trauma, blurring the boundaries between the front line and the home front. Traditional clinical models often face challenges in providing scalable, real-time responses to mass trauma. This study examines “Hero in the Living Room”—a community-based narrative intervention-as a mechanism for processing trauma and promoting resilience during the acute phase of conflict.

**Methods:**

Using a qualitative-phenomenological approach, semi-structured interviews were conducted with 65 participants: 25 active-duty soldiers and 40 civilians who hosted or attended the sessions. Data were analyzed using Reflexive Thematic Analysis.

**Results:**

The findings reveal a dynamic of Reciprocal Healing underpinned by three psychosocial mechanisms: (1) *Narrative coherence*: soldiers utilized the audience to organize fragmented memories into a linear narrative, gaining social validation; (2) *From helplessness to agency*: civilians transitioned from passive spectators to an “Active Container”, engaging in a functional transformation of the domestic space into a therapeutic setting; and (3) *The Mutual defense alliance*: an emotional exchange occurred wherein soldiers provided a sense of physical security/meaning, while the community offered psychological security and normalization.

**Conclusions:**

The study suggests that community-based narrative interventions serve as effective complementary tools to clinical therapy. By relocating trauma discourse from the clinic to the living room, the model bridges the civil-military gap and fosters collective resilience in times of national crisis.

## 1 Introduction

The “Swords of Iron” war created a trauma-saturated social reality in which the military experience and the civilian experience merged into a single crisis texture of *mass trauma*. Entire communities found themselves grappling with fear, uncertainty, loss, and a sense of existential vulnerability, alongside soldiers and combatants engaged in long, arduous hours of fighting, rescue, and defense. This reality posed a significant challenge to traditional clinical models, which often struggle to provide scalable, immediate responses for processing traumatic experiences among broad populations.

Within this reality, social and community “bottom-up” initiatives emerged, designed to bridge the gap between the battlefield and the civilian sphere, enable the processing of difficult experiences, and strengthen the sense of solidarity and resilience. One of the prominent initiatives that developed against the backdrop of the war is “Hero in the Living Room” (*Gibor BaSalon*)—an intimate encounter between soldiers who experienced the war and a civilian community seeking to understand, listen, and give space to the combatant's narrative.

The “Hero in the Living Room” initiative typically takes place in the domestic space, whether in a family living room, a community center, or private hosts' homes. A central characteristic of the meeting is the intimate and informal atmosphere, marking the shift of discourse from the clinical-sterile space to the domestic space. The meeting allows soldiers to tell their personal stories, reflect on experiences of heroism, fear, resourcefulness, and camaraderie, and share emotional depth that, in most cases, does not find expression in the broader public discourse.

The presence of unit members in these meetings transforms the event not only into a public discussion but into a kind of community ceremony of *Mutual Recognition*, granting the community's recognition of the soldiers and the soldiers' recognition of the community as a supportive psycho-social infrastructure for emotional containment and social validation. The “Hero in the Living Room” (Gibor BaSalon) initiative is a grassroots, non-governmental project that emerged spontaneously from civil society during the conflict. Rather than a formal, top-down clinical program, it functions as a flexible community model where local hosts or volunteers initiate *ad-hoc* encounters. These sessions are not led by clinical therapists but are facilitated through an informal, peer-to-peer approach, prioritizing an organic social atmosphere and shared human experience over professional hierarchy and formal clinical protocols.

This reciprocal relationship benefits both sides: while soldiers gain an opportunity to process war experiences, build an empowering narrative, and gain social recognition, the hosting community gains the creation of collective meaning, anxiety reduction through unmediated proximity to the circle of combat, and the *Restoration of Agency* (1, 7).

In this sense, “Hero in the Living Room” represents a unique form of “Community Trauma as Healing,” a process in which the very act of sharing, listening, and reciprocity produces therapeutic mechanisms that are not institutional but human, spontaneous, and profound. The meeting is not merely a stage for storytelling but a space of repair, where the possibility of talking about trauma is rebuilt without being pushed to the margins of discourse.

Recent research literature indicates that communities exposed to national trauma events can develop unique resilience mechanisms by creating shared spaces of meaning and belonging (2, 3).

When a community mediates difficult experiences through civic ceremonies, informal gatherings, and intergenerational discourse, processes of Communal Trauma Processing as Healing develop, reducing the sense of loneliness and strengthening trust in the surrounding human systems (4).

In the Israeli context, where combat and civilian life are intertwined, this initiative serves as a distinct example of how the community generates support and resilience mechanisms in real time.

This study seeks to examine in depth the unique contribution of the “Hero in the Living Room” initiative to both sides of the encounter: the soldiers arriving from the war to tell their story, and the community, present in the living room, listening, participating, and sometimes becoming part of an internal meaning discourse developing within the soldier. The study emphasizes that the dynamics occur within a “Shared Traumatic Reality,” where both the speaker and the listener are exposed to the threat. In many meetings, the very presence of unit members, partners in the experience, creates a safe, strengthening, and sometimes emotionally constitutive space for the soldier. Thus, “Hero in the Living Room” is not just another community gathering but a complex interaction of mutual support where both sides undergo a process of processing, recognition, and healing.

The study focuses on understanding the psycho-social processes generated within these meetings and examines the experience through the eyes of participants on both sides of the initiative.

This qualitative study is based on 65 in-depth interviews: 25 soldiers-including both individual combatants and organic unit members-who participated in the initiative and chose to share their stories.

The objectives of the study are to examine:

1. How do soldiers experience the “Hero in the Living Room” meetings, and how do they perceive the contribution of these meetings to processing the experience and empowering personal resilience?
2. How do civilian participants experience the meeting, and what is its contribution to community resilience, understanding of war experiences, and the sense of meaning?
3. How does the dynamic between soldiers, their unit members, and the community create a shared healing space-constitutive, strengthening, and sometimes therapeutic-for everyone?

By mapping these processes, the study seeks to contribute to the understanding of community models of resilience and healing, and to shed light on a significant social initiative developing in Israel during an era of continuous national crisis.

This study presents a unique contribution to the research literature in the fields of trauma, personal and community resilience, and the processing of war experiences, by examining a new social-community model of “Hero in the Living Room” meetings. This model is characterized by several unique aspects that distinguish it from classical models:

(A) Processing in the acute phase: soldiers in “real-time”—the study participants are combatants who have experienced the events recently (and are sometimes still in active service), rather than war veterans processing events from a distance of years (Retrospective). This fact allows for an examination of trauma processing in close proximity to the events.
(B) Direct encounter with the civilian community: the meeting takes place face-to-face, in an intimate space, thereby allowing a direct connection between the military experience and the community response, bridging the “civil-military gap.”
(C) Informal character and domestic/community space: defining the living room as a home gathering creates an open and empathetic atmosphere, allowing for emotional sharing and the tightening of social bonds, unlike the hierarchy characterizing clinical therapy.
(D) Emphasis on discourse, sharing, and mutual resilience: the meeting serves as a healing mechanism for both the soldiers and the community, enabling the building of personal and community resilience within a shared space. This is a model of *Reciprocal Healing*.

Therefore, this study fills a significant research gap, as meetings of this type have not yet been systematically researched while examining the mutual influence of the soldier's and the community's experiences on trauma processing, resilience strengthening, and the generation of collective meaning.

## 2 Literature review

This literature review presents the theoretical and empirical foundation for the study of the *Hero in the Living Room* initiative, focusing on how grassroots community initiatives serve as a response to ongoing national trauma. The review is organized into three central axes corresponding to the research questions:

1. The psychosocial axis, focusing on the re-constructing of personal narratives by the soldiers.
2. The sociological axis, which analyzes the shifts in collective memory and the role of informal encounters as a mechanism for bridging the civil-military gap.
3. The applied axis, which establishes the theory of ‘Communal Trauma Processing as Healing' and the model of reciprocity formed between the combatants and the community.

### 2.1 The psycho-social axis: trauma processing, narrative, and personal growth

This process of narrative processing aligns with the foundational principles of Narrative Therapy (5), emphasizing the “re-authoring” of life experiences as a vehicle for transformative change (55). Contemporary applications of this framework among combatants highlight the critical role of narrative processing in facilitating moral repair and enhancing resilience (6, 7). Personal narratives allow soldiers to re-construct their experiences, a cognitive and emotional effort that can be decisive for trauma processing and psychological healing (8). In this context, Judith Herman (9) argues that recovery from psychological trauma is fundamentally a social process; she emphasizes that healing cannot occur in a vacuum, but rather requires a ‘community of witnesses' and social recognition that restores the survivor's sense of belonging and human connection.

The ability to create a structured and meaningful story from traumatic experiences-*narrative coherence*-is critical for coping with PTSD. Soldiers often struggle with this coherence due to the emotional and cognitive dissociation that occurs during traumatic events (10). However, writing and sharing stories help soldiers gain control over their memories and emotions (8), where the integration of positive emotional expressions in trauma narratives is linked to lower levels of psychological distress (57). The content of trauma narratives, particularly the use of positive and negative emotion words, reflects emotional processing more than the grammatical structure of the narrative itself (11).

The theoretical foundation for understanding the living-room encounter is rooted in the concept of “testimony.” As Felman and Laub (12) argue, a traumatic event is characterized by a “lack of witnessing” in real-time, as the intensity of the event prevents the subject from cognitively containing it. Consequently, healing depends on the existence of a “witnessing address”—a listener who enables the survivor or soldier to become a witness to their own story. However, LaCapra (13) cautions against the tension between “working-through” and “acting-out.” While constructing a coherent narrative is a reparative tool that grants mastery over memory, there is a risk that coherence may lead to the “sanitizing” of trauma. Such a process might blur the sharp edges of guilt and moral injury to make the testimony more palatable for the audience.

### 2.2 Post-traumatic growth (PTG) and identity shift

Narratives contribute to personal growth by assisting soldiers in re-constructing their identities and experiences (14). This process, rooted in the “re-authoring” principles of Narrative Therapy (5), allows veterans to transform fragmented memories into a coherent story of agency. Soldiers who narrate their experiences in terms of personal growth and agency report improved mental health outcomes (7). Autobiographical storytelling facilitates the re-constructing of traumatic memories and supports a sense of autonomy, which is vital for completing one's life narrative (15).

In this context, narratives of heroism can aid the psychological resilience of military personnel by allowing them to process and articulate their experiences (1, 16). Furthermore, they assist veterans in moving from trauma-saturated narratives to those rich in possibilities and meaning (17). However, military culture, which often emphasizes stoicism and toughness, may inhibit emotional expression and make it difficult for soldiers to engage in narrative therapy (58). Additionally, narratives of *Moral Injury* require careful handling to transform internal monologs into constructive dialogues (18).

### 2.3 The sociological axis: shifts in memory, collective identity, and media representation

This section situates the initiative within the sociological context of memorial ceremonies and examines the contribution of heroism stories to the listening community.

#### 2.3.1 Stories of heroism as a bridge to mutual understanding and solidarity

Soldiers' stories of heroism serve as a bridge between the military and civilian worlds, offering insights into the complexity of military life. These narratives help civilians understand the complexity of war-related trauma. Narrative interventions (such as film or verbal storytelling) improve soldiers' perception of civilians as a supportive and receptive entity, thereby developing a supportive environment that facilitates reintegration into society.

Listening to soldiers' stories without interruption or judgment produces significant emotional relief for the narrating soldiers (54). For civilians, this listening enables a complex and multidimensional understanding of the reality of war, beyond the simplistic narratives often prevalent in the media. Narratives about shared history and values help military personnel feel a sense of belonging to the community and bridge civil-military cultural gaps.

#### 2.3.2 From “Zikaron BaSalon” to “Hero in the Living Room”: evolution of the commemoration model

The transition from private distress to collective meaning in this initiative is better understood through Maurice Halbwachs' (19) concept of “social frameworks of memory”. Halbwachs argues that individual recollections are always mediated by the social groups to which individuals belong, providing a collective structure for personal narratives. On this basis, the conceptual foundation of the “Hero in the Living Room” (Gibor BaSalon) initiative is rooted in the “Memory in the Living Room” (Zikaron BaSalon) project, which has transformed Holocaust commemoration in Israel over the last decade (53). The original model was created as an alternative to formal state ceremonies, arising from the need for an intimate space that allows for direct testimony from survivors before a small audience. The transition from the national stage to the private living room facilitates the breaking of barriers between the “witness” and the “audience,” transforming memory from a passive act into an active act of participation and dialogue. Subjective reports from this study's participants indicate that this dynamic replicates in encounters with soldiers, dissolving the traditional partitions between the military and civilian spheres.

The “Hero in the Living Room” initiative adopts this platform but performs a “functional displacement” (Functional Displacement): rather than historical testimony about distant past events, the model is now used to process a current, “bleeding,” and ongoing event by young combatants. The use of the same “Setting” signals to participants that this is a space of “civil sanctity” (Civil Sanctity)—a protected environment separated from the mundane that allows for deep listening. Unlike the commemorative focus of the original model, the primary goal here is to create a tool for trauma processing and resilience in real-time. Utilizing the familiar “Zikaron BaSalon” framework grants the new initiative immediate social legitimacy and a “cultural shortcut”; it defines the unwritten rules of the ceremony for the community-respect, containment, and avoidance of judgment-which are intuitively derived from collective memory.

In this study, “heroism” is treated not as a self-evident trait or a mere physical act, but as an analytical and social construct predicated on the interplay between duty (Duty), sacrifice (Sacrifice), and social recognition (Recognition) (20, 21). Current research suggests that in “post-heroic” societies (Post-heroic societies), the definition of heroism is often contested (22). While public discourse may lean toward simplistic militaristic glorification, the individual soldier's experience is frequently characterized by the tension between professional courage and “moral injury” (Moral Injury) (18, 23). Therefore, heroism is defined here as “narrative authority” (Narrative Authority) reclaimed by the individual (21). This allows for a complex representation that includes vulnerability and ethical dilemmas, replacing the binary “hero/victim” label with a multidimensional human narrative. This conceptual framework facilitates an examination of how community recognition in the “Hero in the Living Room” initiative serves as a mechanism for processing the emotional burden associated with the heroic identity.

#### 2.3.3 Narratives and social structures: myth, gender, and media representation

Narratives of heroism and courage are an integral part of the cultural and historical fabric, essential for formulating national and community identity. For instance, the mythological narrative of ANZAC soldiers in Australia became a central pillar of national identity (24). The narrative of patriotic sacrifice continues to be a fundamental element in the formation of national identity.

However, the construction of military heroism is highly complex, as it intersects with gender issues and reflects broad social expectations (25). While media representations often lean toward stereotypical narratives of either heroism or trauma (26, 26), the intimate encounter within “Hero in the Living Room” provides a unique space to dismantle these binaries. Within the domestic sphere, combatants-both men and women-can construct a narrative of heroism that does not rely solely on stoic toughness but instead integrates vulnerability and emotion, thereby challenging traditional gendered military stereotypes.

Furthermore, heroism stories may at times overshadow other narratives, such as those of suffering and exploitation (27), highlighting the constant tension between the myth of heroism and the harsh reality of war (16, 27). The concept of “post-heroic societies” indicates that modern societies often struggle to reconcile the reality of military violence with their core values (22). In the Israeli context, “Hero in the Living Room” serves as a crucial bridging mechanism for this tension; it enables the community to process the difficult reality of war not through distant, mythologized figures, but through direct, humanized interaction. This allows veterans to reclaim their narrative authority, resisting the simplification or politicization of their experiences while fostering a new shared language between the battlefield and the civilian sphere.

#### 2.3.4 Challenging stereotypes and reclaiming narrative authority

Storytelling allows soldiers to reclaim their symbolic authority and develop individual versions of their identity, thereby dismantling the binary stereotypes of “the Hero” vs. “the Victim” (21). By sharing their stories, veterans can resist politicization and the simplification of their experiences (59). Structured frameworks (such as the Human Library) provide a safe environment for storytelling, which aids in creating connections between civilians and soldiers and alleviates negative psychological symptoms (28).

### 2.4 The applied axis: community trauma, resilience, and reciprocal healing

This section is the theoretical core of the study, examining the bidirectional dynamics between the soldier and the community as the creation of a shared healing space.

#### 2.4.1 Community resilience as a product of shared narrative

Stories of heroism and bravery serve as a pivotal element in constructing community resilience, as they forge a collective consciousness and reinforce a sense of shared purpose (29). Such narratives of resilience transcend the individual soldier, encompassing entire communities and reflecting adaptive processes that bolster a community's capacity to recover from adversity and crisis (30). Moreover, these narratives facilitate collective healing by involving communities in the “narrativization” of trauma-a proactive mode of processing that nurtures resilience and fosters communal post-traumatic growth (31, 56).

#### 2.4.2 The active container: civilian transformation in narrative sharing

Storytelling initiatives, such as the R.E.A.D.S. program for soldiers, clarify how the practice builds resilience and community among combatants (32). Soldiers' stories of sacrifice and resilience serve as catalysts for social healing and intergenerational solidarity by establishing a psycho-social infrastructure that incorporates emotional containment and social validation (33). This narrative identity, emphasizing collectivist values, acts as a protective social factor enhancing community cohesion (33). In military systems (such as New Zealand), narratives are used to encode resilience and recovery by integrating cultural perspectives on well-being (34). The camaraderie (*Re'ut*) among soldiers-highlighted in their stories-creates a critical support system that fosters resilience (60). Furthermore, the healing derived from communal trauma processing reinforces civil solidarity and public support, which often rely on the belief in soldiers' commitment to the mission (20).

#### 2.4.3 Challenges in dialogue and the need for sensitivity

While soldiers' stories of heroism can assist in trauma processing, an inherent tension exists between the need to remember and the desire to forget traumatic experiences, complicating the narrative process (16, 35). This tension often reflects the conflict within “post-heroic societies” struggling to reconcile national heroic ethos with modern liberal and individualistic values (22). Furthermore, the idealization of heroism can exacerbate moral injury by reinforcing cultural norms that encourage masculine stoicism while ignoring the detrimental effects of military service (23, 58). This complexity highlights the need for culturally informed approaches to narrative therapy that allow combatants to reclaim their symbolic authority over their personal stories (18, 21). Shared narratives between soldiers and civilians assist in treating moral injury by challenging gender stereotypes and encouraging society to move beyond simplistic hero narratives toward recognizing complex human agency that integrates vulnerability and moral dilemmas (23, 25).

In conclusion, the literature review demonstrates that the ‘Hero in the Living Room' initiative is built upon a solid theoretical foundation encompassing trauma psychology (PTG), narrative processing (sociology of memory), civil rituals, and communal healing. Sources confirm that sharing personal narratives is an essential tool for personal rehabilitation and strengthening national resilience, as these stories create a shared vision and define values (29). However, a significant research gap remains regarding the systematic and qualitative analysis of reciprocal interaction in these encounters. While the literature identifies the benefits for each side individually (emotional relief for the soldier, understanding for the community), the transactional dynamics and the impact of the presence of unit members on mutual healing have yet to be explored in depth. This study, based on in-depth interviews, will fill this gap and contribute to the understanding of the ‘Communal Trauma Processing as Healing' model in real-time.

## 3 Methodology

### 3.1 Research design and rationale

The present study was conducted using an Interpretative Phenomenological approach, anchored in a social-constructivist epistemology. This approach focuses on understanding the meaning of subjective experiences through the participants' own perspectives (40). This choice was intended to ensure methodological integrity, creating a direct alignment between the research questions and the investigation of the participants' “lived experiences.” While the phenomenological framework guides the inquiry into the interviewees' inner worlds, the actual analysis was carried out through Reflexive Thematic Analysis (36). This integration allows for an in-depth phenomenological exploration of personal experience alongside the systematic identification of cross-cutting patterns and meanings (themes) in the data.

The qualitative approach allows for the discovery of emotional, communal, and value-based layers of the human experience, creating a rich and multi-dimensional picture of coping with combat and bereavement. Qualitative studies indicate their contribution to understanding deep emotions, emotional processing, and community coping mechanisms. The choice of this method is consistent with the research objective: to uncover the personal and social meanings assigned by soldiers and civilians to the shared encounter and the ways they processed the war experience within the domestic space.

For data collection, semi-structured in-depth interviews were used, designed to allow free yet focused conversation while maintaining a structure that enables comparison between interviewees (37). Such interviews have been found effective in researching sensitive emotional topics and promoting authentic discourse in situations of trauma, heroism, and meaning (38). The process of building the interview guide was based on an extensive literature review and included an initial pilot of several experimental interviews. The purpose of the pilot was to test the clarity of the questions, their level of cultural and emotional sensitivity, their logical arrangement, and the resilience of the interviewees over an extended interview period (37). Following the pilot, specific changes were made, mainly in the phrasing and order of the questions, to improve the level of engagement, accuracy, and emotional containment during the conversation. This process contributed to the validation of the research tools and increased the trustworthiness of the findings, as required by professional standards for qualitative reporting.

### 3.2 The “Hero in the Living Room” initiative: structure and procedures

Operationally, the meetings are coordinated in advance through social platforms or community coordinators and follow a consistent social structure lasting between 90 to 120 min. The sessions are divided into four sequential stages: (1) Gathering and Opening, where the host defines the boundaries of the space, emphasizing discretion and non-judgmentalism; (2) narrative Testimony, involving the soldier's continuous story (approximately 45 min); (3) community Dialogue, featuring questions and reflections from the audience to foster social validation; and (4) closing and Reflection, allowing for initial processing and a transition back to routine. Hosts receive a preliminary briefing on identifying signs of emotional flooding or dissociation in real-time, enabling a proactive pause if necessary. Following the session, the host conducts a brief debriefing with the speaker to ensure their emotional well-being and, if required, activates the initiative's professional “Safety Net,” providing a clear referral path for further emotional support.

### 3.3 Study population and sampling

The research population consisted of 65 participants (*N* = 65) in the “Hero in the Living Room” initiative, divided into two core groups: 25 male and female soldiers who personally experienced combat in the “Swords of Iron” war and participated as speakers, and 40 participants from the civilian community who hosted or attended these meetings. The sample included both lone soldiers and members of organic fighting units, a distinction that allows for the examination of reciprocal healing processes in diverse military and social contexts. Participants were selected using purposeful sampling, with the intent to include a wide range of ages, genders, service types (mandatory/reserve), and community backgrounds, aiming to illuminate the diversity in personal and communal responses to the encounter (39) while addressing the civil-military cultural gaps between the military system and the civilian community.

This sampling method is considered particularly suitable for qualitative research, where the emphasis is placed on the depth and context of experiences rather than statistical generalization.

Recruitment procedure: participants were recruited through snowball sampling. To locate the initial interviewees (seeds), initial contact was made with community coordinators of the “Hero in the Living Room” project in the relevant areas, who served as “gatekeepers” and mediated the outreach to the first hosts and speakers. Following these interviews, participants were asked to refer the researcher to additional soldiers or hosts who took part in the initiative. To prevent pressure to participate, direct contact with interviewees was made only after initial consent was obtained, and interviews were coordinated after the conclusion of the community meeting rather than during it.

The interviews took place during the first 6 months of the war, according to the participants‘ choice, in convenient locations such as the interviewees' homes or private digital spaces (Zoom), and at times that allowed for a relaxed environment and space for personal and emotional discourse. This consideration was intended to allow for maximum openness and a sense of security, especially given the sensitivity of the topic of trauma and personal disclosure (40). Each interview lasted between 60 to 90 min and was fully transcribed by the researcher.

Table 1 presents the demographic and occupational characteristics of the soldiers participating in the study. The table allows for an understanding of the sample composition demographically and professionally, reflecting the diversity and representativeness of the study participants.

**Table 1:** Demographic, military, and service characteristics of the combatant group (*N* = 25).

**Variable**	**Category**	**Number of participants (*N*)**	**Percentage (%)**
Age	20–24	12	48%
	25–29	8	32%
	30 and above	5	20%
Gender	Men (combatants and combat support)	19	76%
	Women (combatants/combat support at the front)	6	24%
Service status	Regular (mandatory/permanent)	10	40%
	Reserves (over 60 days)	15	60%
Recruitment status at interview	Mobilized (during active service/Short leave)	18	72%
	Released (break or release from reserves)	7	28%
Type of combat exposure	Active combat/direct encounter	18	72%
	Rescue and evacuation of wounded under fire	7	28%
Occupational status	Student/academic	13	52%
	Self-employed/free profession	12	48%

An examination of the data in the table shows that the group of soldiers is mostly composed of a young population (80% in the 20–29 age range). A critical finding for understanding the research results is the military status at the time of the interview: 72% of the participants (18 soldiers) were still on active duty (regular or reserve) during the interview. This fact emphasizes that the narrative processing occurred “on the move”, within the chaotic reality of the war, rather than from a distance of time.

Based on the data in Table 2, it should be emphasized that the civilian population in this study (*N* = 40) does not constitute a neutral or external “audience.” Analysis of demographic characteristics reveals that participants are within a “Shared Traumatic Reality”: 55% of them are parents or first-degree relatives of soldiers at the front, and all were exposed to the existential threat of October 7th. Therefore, the investigated encounter is not dichotomous (a healthy caregiver vs. an injured patient), but rather an encounter between two subjects suffering from distress at different levels: the soldier faces direct trauma (exposure to death), while the civilian faces secondary trauma, constant anxiety, and a sense of helplessness. This understanding is critical for analyzing the dynamics of reciprocal healing.

**Table 2:** Demographic characteristics and involvement of the civilian community group (*N* = 40).

**Variable**	**Category**	**Number of participants (*N*)**	**Percentage (%)**
Age	30–45	18	45.00%
	46–60	17	42.50%
	61 and above	5	12.50%
Education	Bachelor's degree and above (academic)	30	75.00%
	Post-secondary education (non-academic)	10	25.00%
Role in encounter	Host (opened their home)	15	37.50%
	Participant	25	62.50%
Proximity to trauma (shared reality)	First degree relative to a soldier at the front (parent/spouse)	22	55.00%
	Evacuee/resident of the Gaza envelope	5	12.50%
	No direct personal involvement	13	32.50%

### 3.4 Research tools

The primary research tool in this study was a semi-structured in-depth interview, chosen for its ability to allow a flexible yet systematic exploration of participants' personal experiences, perceptions, and emotions regarding sensitive issues (37, 38). The interviews were conducted in the participants' native language (Hebrew) and were subsequently transcribed and translated into English, ensuring semantic equivalence to preserve the nuance and accuracy of cultural and emotional terms.

The data collection process continued until thematic saturation was achieved. During the interviews, participants were asked, among other questions: how did the direct encounter affect your sense of personal processing? What emotional reactions arose during sharing in front of an audience? How was the connection between the soldier and the community perceived? Furthermore, sub-questions were included to explore the “dyadic self” (the interaction between soldier and listener), the physical/somatic sensations experienced during storytelling, and the tension between emotional exposure and the military ethos of stoicism and toughness.

Additionally, specific prompts were included to explore the construct of heroism, such as: “How did it feel when the hosts or audience addressed you as a ‘hero'?” and “Did you experience a gap between the public ‘hero' label and your internal feelings of fear or vulnerability?”

### 3.5 Procedure and data analysis

Interview transcripts were analyzed using qualitative content analysis based on the thematic analysis model, utilizing inductive open coding to identify patterns, themes, and recurring narratives (36, 41). The analysis was performed iteratively according to Braun and Clarke's six steps: (1) Familiarization with the data; (2) Generating initial codes; (3) Searching for themes; (4) Reviewing and refining themes; (5) Defining and naming themes; and (6) Producing the report.

The coding process was conducted while maintaining standards of reliability, consistency, and transparency (42). To maintain the reliability of the analysis and reduce researcher bias due to solitary coding, several key steps were taken (42, 61):

1. Member checking: following initial analysis, summaries of the central themes and representative quotes were presented to several key participants (approximately 20% of the sample) for verification and confirmation that the developed themes accurately reflected their experience.
2. Audit trail: detailed documentation of all analysis stages, including examples of raw coding, allowing for tracking and full transparency.
3. Peer debriefing: holding discussions with a colleague researcher to examine the coding, critique the interpretation, and reduce subjective bias.

The analysis involved repeated reading of all interview transcripts, identification of significant statements, assigning them to conceptual categories, and subsequently formulating central themes. In this process, a balance was maintained between open reading of the findings and reference to theoretical anchors related to grief, Post-Traumatic Growth (PTG), collective memory, and community resilience (43).

In the coding process, “heroism” was operationalized as a contested narrative rather than a self-evident label. Specific codes were developed to identify how heroism was negotiated as either a self-description, a social label, or a resisted media discourse, addressing the tension between societal expectations and the participants' complex emotional reality, including resistance to simplistic militaristic glorification.

To provide a transparent representation of the findings, a theme-frequency mapping was conducted. The unit of analysis was the individual participant, where a frequency count represents the number of participants who explicitly discussed a particular theme. In cases of thematic overlap, each conceptual dimension was coded independently to ensure comprehensive coverage without inflating the relative weight of the themes.

### 3.6 Research ethics—clinical commitment and sensitivity

The study's design and execution were approved by the relevant institutional ethics committee. The research was conducted in accordance with established ethical principles for qualitative research, with a specific emphasis on protecting populations in crisis situations. Participation was entirely voluntary, following a full explanation of the research goals, data collection stages, and the right to withdraw at any time (37). To ensure the autonomy of the combatants and prevent command-related pressure, the recruitment process was conducted independently and outside the military chain of command, with coordination through relevant casualty departments serving solely as a mediating factor.

Each participant signed an informed consent form, and full confidentiality and anonymity were guaranteed. Due to the sensitive nature of the study, rigorous measures were taken to ensure discretion, privacy protection, and a sensitive approach to mental health aspects (42). All interviews took place in locations chosen by the participants to create a “Holding Environment”—a safe, inclusive, and non-judgmental space conducive to narrative trauma processing.

Interview transcripts were fully anonymized. The researcher possesses specialized training and experience in managing discourse in crisis and trauma situations. These skills enabled the researcher to identify signs of distress and provide initial emotional support when necessary (36). However, a clear distinction was maintained between providing point-of-need emotional support during the interview and formal therapeutic intervention. For participants exhibiting significant distress, a “referral path” was pre-established, providing a list of specialized mental health services for continued professional assistance. The researcher ensured cultural adaptations and operated from a deep commitment to maintaining a respectful and safe environment (43).

### 3.7 Reflexivity and researcher positionality

The researcher is a psychotherapist, mental health first responder, and expert in trauma and complex trauma, involved in accompanying communities during times of crisis. This position-both as a clinician with deep professional knowledge and as a resident of the country sharing the collective traumatic reality with the participants-required extra caution. On one hand, clinical expertise allowed for the identification of nuances in emotional processing. On the other hand, there was a need for constant awareness to prevent the projection of prior professional knowledge onto the interviewees' narratives. To address this challenge, the researcher maintained a Reflexive Journal and performed a process of “Bracketing” (suspending judgment) to ensure listening that was as clean as possible to the participants' voices.

### 3.8 Trustworthiness and rigor

To ensure the quality of the study and the accuracy of the findings, methodological steps were taken in accordance with the criteria of Guba and Lincoln (44):

- Credibility: a process of Member Checking was performed. A draft of the findings was presented to a sample of participants (soldiers and civilians) to verify that the interpretation faithfully reflects their subjective experience.
- Dependability: a detailed “Audit Trail” was maintained, including interview transcripts, field notes, and coding tables, allowing for transparency in the conclusion-drawing process.
- Confirmability: a Peer Debriefing procedure was conducted for critical analysis of the themes and to minimize subjective biases.

## 4 Findings

The analysis of the interviews revealed three central depth axes (Themes) that describe the psychosocial dynamics of the “Hero in the Living Room” initiative. These themes range from the individual level of the soldier, through the experience of the civilian community, to the shared interpersonal space created between them, directly addressing the three predefined research questions (see below). The findings illustrate how the living room encounter transforms from a “legacy story” event into a space for reciprocal trauma processing.

The interviews describe a transformation of the domestic space: crowded living rooms, the smell of home-baked goods mixed with palpable tension in the air, and a tense silence the moment the soldier enters the door. One participant described it this way: “It wasn't like a lecture at a community center. We sat on the carpet, crowded, legs touching legs. When he started talking and trembled a bit, we all held our breath with him.” This “Thick Description” illustrates how the physical and symbolic intimacy of the living room intuitively created a therapeutic setting, allowing for the breaking of emotional boundaries and the transition from formal discourse to a discourse of shared pain.

To reflect the prominence of each theme across the sample and enhance the trustworthiness of the analysis, a mapping of thematic prevalence among the two research groups was conducted. The data presented in Table 3 illustrate the characteristic dynamics of the model: while narrative processing (Theme 1) was central for 88% (22 out of 25) of the soldiers, and the active resilience theme (Theme 2) was evident among 87.5% (35 out of 40) of the civilians, the reciprocity and exchange theme (Theme 3) was highly prevalent in both groups (80%). These patterns suggest the presence of a shared healing space and illustrate the depth of the mutual encounter within the “Hero in the Living Room” framework.

**Table 3:** Frequency of central themes in interviews (*N* = 65).

**Central theme**	**Core meaning**	**Frequency among Soldiers (*N* = 25)**	**Frequency among Civilians (*N* = 40)**
1. “Making Order out of Chaos”	Transition from fragmentary traumatic memory to a coherent narrative; need for social validation to reduce guilt.	22 (88%)	–
2. “From Shock to Action”	Transition from passive viewing to the role of “Active Container”; restoring the sense of Agency.	–	35 (87.5%)
3. “The Mutual Defense Alliance”	Emotional exchange relations: physical security in exchange for mental security, normality, and validation.	20 (80%)	32 (80%)

The thematic mapping presented in Table 3 illustrates that the ‘Hero in the Living Room' encounter is perceived as highly significant and salient for both study groups. While the specific needs of soldiers and civilians diverge-as reflected in the distinct focus of Themes 1 and 2-the pervasive presence of the Reciprocity theme (Theme 3) among eight out of ten participants (80%) underscores the centrality of a shared healing space within their internal worlds. These patterns suggest an interaction that generates a ‘mutual container', transcending isolated individual experiences. In the following sections, each theme is examined in depth through an interpretive analysis of the participants' testimonies.

### 4.1 First theme: “Making Order out of Chaos” and the transition from silence to a coherent narrative

This finding relates to the first research question and focuses on the soldiers' experience. The analysis of the interviews reveals that for 88% of the soldiers (20 out of 25), the living room encounter constituted a turning point in their ability to organize fragmented war memories into a story with a beginning, middle, and end. The soldiers described the preparation for the meeting and the act of speaking before a supportive audience as an action that forced them to “step out of the bubble” and produce narrative coherence. Unlike the operational-technical discourse with their unit members, the discourse with civilians required translating the experience into emotional and clear language.

One of the combatants (R., Combat Reservist) described the need to construct the story for the audience:

“*Inside the battle, everything is noise, explosions, and commands on the radio. There is no time to think about what happened. When I sat down in the living room and started talking, I suddenly understood what we went through. I needed to explain it to them, but actually, I was explaining it to myself. Suddenly the story had logic; it wasn't just a sequence of scary events.”*

Another participant (Y., a member of a rapid response team) reinforced this and described breaking the conspiracy of silence:

“*When I came home to my wife, I was silent for two weeks. I didn't know where to start; I was afraid that if I started talking, I would flood her. Specifically here, in front of strangers who came to listen, the words suddenly arranged themselves. I needed to build the story for them, and it helped me build it for myself. Suddenly I understood what happened there on October 7th.”*

Alongside the cognitive organization, the findings indicate that the “good eyes” of the audience allowed the combatants to release burdens of guilt and shame. This process of Social Validation emerged from non-judgmental listening that provided new meaning to their experience, not only as heroes but as human beings who experienced hardship

A female combatant (D., a paramedic) shared the initial fear and the surprising discovery:

“*I was afraid they would judge us for the decisions we took there. But when I saw people crying with me, nodding, I felt I wasn't alone with this burden. They took a piece of the heaviness from me.”*

A. (a platoon commander) added in this context:

“*There are things you do in war that you aren't proud of, or split-second decisions you regret. When I sat in the living room and told about the dilemma we had in battle, I saw in people's eyes not judgment, but an understanding of the complexity. It validated for me that I am a human being in an impossible situation.”*

Finally, it emerged that the encounter assisted in the transition from traumatic sensory memory to a linear narrative. The living room served as a “Container”, enabling the transformation of sensory impressions into a structured story. As described by S. (a Search and Rescue combatant):

“*Until the meeting, the war was for me mainly smells and noises in my head. It didn't have a beginning and an end. The meeting forced me to create a chronological sequence. To say: ‘First A happened, then B, and finally C.' It turned the chaos into something that can be held, into something that has logic.”*

### 4.2 Second theme: “From Shock to Action”—the community as an active container

This theme answers the second research question and focuses on the experience of the civilian participants. The findings show that prior to the encounter, many in the community experienced severe feelings of helplessness, anxiety, and detachment, fueled mainly by passive consumption of news. The meeting with the soldier in the living room shifted their position from anxious viewers to active participants in the war effort-in the role of “Holders” of the story.

Interviewees described a sense of relief stemming from the direct human encounter, which “brought the war down to earth.” One of the hosts (M., aged 45) described:

“*All day in front of the TV, I felt I was going crazy with worry. When the soldiers entered my home and sat on my sofa, I felt that I was finally doing something. I cannot hold a weapon, but I can hold their hearts. It gave me a tremendous sense of meaning.”*

R. (another host) sharpened the transition from passivity to activity:

“*The news on TV just paralyzed me. I felt I was drowning in anxiety. When I organized the meeting, suddenly I had a task. Arranging chairs, baking a cake, making sure it was pleasant. It sounds trivial, but this doing for the soldiers restored my sense of control over life. I felt I was no longer a victim of the situation.”*

Additionally, it arose that the encounter strengthened community solidarity and gave new meaning to the concept of the “Home Front” (*Oref* ). D. (a pensioner) told about the role of listening:

“*I always thought being a patriot meant waving a flag. In the meeting, I understood that my greatest contribution is simply to be there and listen until the end, even when it's hard to hear. To be a ‘container' for their pain. I left that evening with a feeling that I fulfilled an important role, that I'm not just sitting at home waiting for it to end.”*

Parents of soldiers found the meeting a path to channel their personal worry. G. (a mother of soldiers) shared:

“*I looked at these soldiers and saw my children. It gave me an opportunity to channel my maternal worry into something constructive. Instead of sitting and worrying at home, I gave a real hug to someone who needed it. It filled me with strength for a whole week.”*

### 4.3 Third theme: “The Mutual Defense Alliance”—the domestic space as a shared healing arena

The third and most central theme connects the two parties and answers the third research question regarding mutual dynamics. The findings reveal a mechanism of “reciprocity in security”: while the soldiers provided the community with a sense of physical security (“We are guarding you”), the community provided the soldiers with a sense of mental security (“We are guarding sanity and normality for you”).

The intimacy of the living room nullified the hierarchy and allowed for an eye-level encounter. The soldiers noted that entering a normative home reminded them of the purpose of the fighting. A. (a combat reservist) described it:

“*When I entered the apartment and saw books on the shelf and children in pajamas peeking from the hallway, I remembered what I am fighting for. They thanked us for guarding them, but they didn't understand that they were guarding our sanity. Their home was the reminder that there is a normal world outside waiting for us.”*

A civilian participant (A., aged 55) summarized this dynamic:

“*There was a moment where the soldier went silent, and one of the neighbors simply handed him a glass of water and put a hand on his shoulder. It wasn't ‘hero' and ‘admirers,' it was a mother and son. He guards us at the border, and we guarded him at that moment in the living room. It was an alliance of silence and understanding.”*

The breaking of formal distance in favor of human intimacy was also prominent in the words of N. (a student):

“*In official ceremonies, there is a stage and an audience, there is distance. In the living room, we sat in a circle, it was different... In one moment, when we talked about fear, the boundaries blurred. There was no ‘combatant' and ‘civilian,' there were human beings fearing and hoping together. It was a moment of rare intimacy.”*

M., a reserve officer, phrased the reciprocity sharply:

“*There was an older woman who approached me at the end and said: ‘Thanks to you, I sleep at night.' I answered her: ‘And thanks to your hug now, I will be able to sleep tonight.' It was accurate. We give them physical security, and they give us mental security that someone appreciates and waits for us.”*

This finding highlights the model of “Community Trauma as Healing”-healing did not occur with a therapist in a clinic, but in a space where both sides acknowledged each other's pain and granted it legitimacy.

In conclusion, the findings demonstrate that the interpersonal dynamics created within the “living room” facilitate a process of reciprocal healing, where the emotional needs of both combatants and civilians complement one another. These insights serve as the basis for the discussion in the following chapter, which examines the model's theoretical implications in light of professional literature.

## 5 Discussion

The aim of the study was to examine the psychosocial dynamics formed within the “Hero in the Living Room” initiative and to propose it as a model of “Community Trauma as Healing” during a national crisis. The study findings, as they emerged from the analysis of the interviews, provide a deep understanding of the lived experience of both combatants and civilian participants during the acute phase of the war. These findings reveal a complex picture of Reciprocity: the living room encounter is not merely a one-way arena of testimony, but a transformative space enabling both parties to move from a state of distress and chaos to a state of meaning and action. Below, we discuss these findings in light of professional literature, focusing on the three central axes that emerged in the study.

### 5.1 The soldier: narrative coherence as a tool for trauma processing

The first finding, “Making Order out of Chaos”, revealed that the very need to construct a story for an external audience helped soldiers organize fragmentary memories into a linear and clear narrative. The current findings extend our understanding of narrative processing by highlighting the transition from traumatic sensory memory (smells and noises) to a coherent narrative. In accordance with Ogden's (10) sensorimotor approach, the living room encounter facilitates the transformation of raw bodily impressions into a structured sequence. This process allows combatants to provide meaning and significance to their lived experience from the battlefield, while transforming raw sensory impressions into a coherent narrative, thereby assisting in emotional regulation. This finding is consistent with research literature emphasizing the importance of narrative coherence in trauma recovery. As Warren (8) notes, the ability to gain control over memories through storytelling is crucial for managing stress symptoms. In the current study, soldiers described a process similar to that found by Tappenden et al. (7), whereby phrasing the experience in terms of agency (Agency) and action contributed to a sense of self-efficacy and reduced distress.

However, the current study adds a unique layer regarding social validation (Social Validation). The validation identified in the study constitutes a critical tool for repairing “Moral Injury” (Moral Injury). The transition from a discourse of “hero vs. victim” (21, 26) to a human discourse in the domestic space allows combatants to formulate a “Dyadic Self” (Dyadic Self), where the civilian community serves as a validating “mirror” and an active partner in constructing meaning and bearing the emotional burden. The findings indicate that the intimacy and lack of judgment in “Gibor BaSalon” created a safe infrastructure for expressing moral injury-a phenomenon that usually requires a protected therapeutic space (18).

Participants in the study were not required to “play the hero”; on the contrary, they received social legitimacy to share ethical dilemmas, moments of breaking, and feelings of guilt. This process directly challenges the stoic military ethos (Stoicism) that sanctifies restraint and toughness, allowing for genuine post-traumatic growth through human intimacy that facilitates what is not always possible in the sterile clinical space. In this way, the initiative functions as a space of resistance (Resistance) to simplistic militaristic glorification and allows soldiers to reclaim their ownership over their story (Reclaiming Narrative Authority) as whole human beings facing an impossible reality.

By implementing heroism as an analytic concept, this study demonstrates that the living room encounter serves as a site for negotiating the “hero” label. The findings suggest that while the community often arrived with a pre-constructed social label of heroism, the soldiers utilized the intimate setting to offer a nuanced self-description that integrated vulnerability and moral injury (Moral Injury). This negotiation effectively dismantled the binary “hero/victim” stereotype (21), allowing for a construction of veteran identity that encompasses both professional duty and psychological burden. Thus, heroism in the “Hero in the Living Room” model is not a self-evident trait but a contested narrative authority reclaimed by the combatant in the face of simplistic militaristic glorification.

### 5.2 The community: from passive anxiety to “Active Container”

The second finding, “From Shock to Action,” illuminated the transformation of the civilian community. The literature extensively discusses the impact of soldiers' stories on civilians as a tool for creating empathy and understanding the complexity of war (45, 46). This study strengthens these claims but extends the scope to the concept of active resilience. Study participants described how hosting and listening extricated them from a position of helplessness and granted them an active role in the war effort. This process aligns with Rathnayake's (33) model, suggesting that engagement with narratives of sacrifice and resilience strengthens community cohesion and intergenerational solidarity.

In the context of ‘Gibor BaSalon', the community undergoes a transformation from a passive ‘audience' into an active partner in creating a healing space. This dynamic can be conceptualized through Bion's (47) theory of “Container/Contained”: the community functions as a ‘container' that receives the raw and unbearable sensory impressions of the combatant (‘Beta Elements') and processes them through empathetic listening into a meaningful narrative (‘Alpha Function'). Here, the community is not merely listening but performing an active psychological transformation that enables the conversion of unprocessable horror into logical and sequential thought.

Furthermore, the intimate domestic space creates a ‘Holding Environment' in the spirit of Winnicott's (48) teachings. The living room becomes a protected space allowing the soldier to experience controlled regression and express contents of fear and fragility, knowing that the human environment around him will not shatter in the face of pain but will ‘hold' it for him. Thus, the community's role goes beyond mere listening and becomes an active psychological function (Active Container), enabling the integration of the traumatic experience.

These findings correspond with the “Social Cure” approach (49), which asserts that group belonging and identification serve as a psychological resource protecting against stress. The current study emphasizes that the civilian transformation is not only personal but represents a reconstruction of Collective Efficacy in the face of shared trauma. The civilians' transition to a mode of “doing for the soldier” allows them to regain the sense of control and meaning lost on October 7th, thereby strengthening overall social resilience.

### 5.3 The shared space: the mutual defense alliance and bridging the gap

The central contribution of this study lies in the third theme-Reciprocity. Professional literature discusses the “Civil-Military Gap” and the difficulties of soldiers' reintegration into a “post-heroic” society that struggles to digest violence (22, 50). The “Gibor BaSalon” initiative is revealed in this study as a critical bridge narrowing this gap in real-time.

The findings point to an Emotional Exchange. To illustrate this unique dynamic, a circular model of resilience resource exchange can be drawn:

1. From the Soldier to the Community: the soldier, by his very presence and story, grants the community physical security (“I am your protective shield”) and meaning (sacrifice for the common good). This gift heals the civilian's sense of helplessness and restores a sense of protection (in alignment with 20).
2. From the Community to the Soldier: the community, through hosting and listening, grants the combatant normality (“Home”), Validation for his feelings, and mental security. This gift heals the combatant's sense of alienation and guilt.
3. In the Center: in the encounter between them, “The Third Space” is created-the shared healing space.

The soldiers provide the community with a sense of physical security and national meaning (as argued by 20, regarding the link between public support and soldier motivation), while the community provides the soldiers with “normality,” a civilian anchor, and social recognition. This is a living example of the claim by Mamon et al. (51) that narrative interventions improve soldiers' perception of civilians as a supportive factor. Moreover, the study shows how a renewed collective identity is formed (29), where the boundaries between “Front” and “Home Front” blur in favor of a shared narrative of coping. Sharing stories within the domestic space-rather than in alienated state ceremonies-is what enables mutual healing and the creation of a shared “We” during a crisis.

In conclusion, the “Gibor BaSalon” initiative demonstrates how a community-based narrative intervention can serve as a powerful mechanism for mutual healing. While existing literature often focuses on clinical treatment for soldiers or community resilience separately, this study shows how the two dimensions are intertwined. The soldier's healing depends on the community's ability to listen, and the community's resilience depends on its ability to take part in the soldier's narrative.

### 5.4 Ethical reflexivity and narrative boundaries

The findings of this study necessitate a reflexive examination of narrative boundaries. The consensus on “avoiding political judgment” within the “Hero in the Living Room” initiative functions simultaneously as a containment mechanism and a narrative boundary. While this depoliticization enables an intimate connection within the “shared traumatic reality” of Israeli society, it also forecloses the impact of war on populations outside the in-group. As Rothberg (52) suggests through the concept of the “implicated subject,” the soldier is not merely a victim of trauma or a hero of action, but a subject operating within a broader conflict context. Acknowledging this limitation is essential for interpreting the constructs of heroism and reciprocity in this study; the initiative enables the expression and validation of the lived experience within a protected and containing space, even if it is politically limited by definition, as the “narrative repair” occurs within a framework that pre-defines its moral horizons.

## 6 Summary and conclusions

This study sought to examine the “Gibor BaSalon” (Hero in the Living Room) initiative not as a commemoration event, but as a real-time psycho-social intervention. The analysis of the interviews reveals that the initiative constitutes an innovative model of “Community Trauma as Healing.” The central conclusion arising from the study is that moving the trauma discourse from the sterile clinic to the domestic living room creates a unique mechanism of therapeutic reciprocity: the soldier is not merely a “patient” in need of unburdening, and the community is not merely a passive “audience.” An unwritten alliance is formed wherein the soldier grants the community a sense of security and meaning, and the community grants the soldier validation, containment, and an anchor for normality.

The study demonstrates that breaking the partitions between the “Front” and the “Home Front” is vital for building national resilience. While traditional clinical models focus on the individual, this model proves that the healing of the individual (the soldier) is intertwined with the healing of the group (the community). The soldier's ability to create a coherent narrative depends on the existence of an attentive community “container,” and the community's ability to recover from the shock depends on its ability to take an active part in the combatant's story.

### 6.1 Study limitations

This study has several limitations that should be considered:

1. Sample bias (self-selection): participants chose to partake in the initiative of their own volition. This population may inherently possess high ego strength and resilience. As noted by the reviewer, this constitutes a significant limitation as it excludes the “avoidant group”-combatants suffering from acute post-traumatic symptoms for whom social exposure might be perceived as threatening or overwhelming. Consequently, the findings reflect the model's efficacy for a specific subgroup, and future research is required to examine the model's suitability for populations with higher levels of distress.
2. Temporal context: the study was conducted “on the move,” during the height of the war. The participants' perspective is acute and immediate. A longitudinal study might present a different picture regarding the long-term effects of exposure.
3. Cultural context: the findings are deeply rooted in Israeli culture, characterized by high solidarity and blurred boundaries between the military and society. Caution should be exercised when attempting to generalize these findings to more individualistic cultures.
4. Researcher bias and single coding: since the study was conducted and coded by a single researcher with a clinical background, there is a potential for subjective bias in thematic interpretation. Although reflexivity measures were employed, the lack of additional coders (inter-rater reliability) remains a methodological limitation. Future studies should incorporate multiple coders to enhance the rigor and reliability of the analysis.

### 6.2 Applied recommendations

Based on the findings, it is recommended that mental health professional, the military, and welfare systems consider the following steps:

1. Adopting the model as a supplementary tool: recognizing initiatives such as “Gibor BaSalon” as a supplementary intervention tool (integrated alongside clinical treatment) for processing combat experiences, especially during the initial stages of returning to civilian life.
2. Developing a structured protocol and training community facilitators: creating training modules for hosts and community leaders to provide them with basic “mental health first aid” tools, the ability to identify states of dissociation or acute distress in the speaker in real-time, and defining clear courses of action for extreme situations. It is recommended to develop a standardized facilitation protocol to ensure that the domestic space remains safe and protected for both the soldiers and the participants.
3. Establishing a professional “Safety Net”: creating a support network of mental health professionals who will be available to provide consultation and guidance to hosts, serving as a direct referral address in cases where unusual emotional flooding requiring clinical intervention is identified.
4. Encouraging diverse narratives: creating legitimacy for sharing stories that are not only “heroic” in the classical sense but also include coping with fear, pain, and moral dilemmas, as part of the process for preventing Moral Injury.
5. Expanding the scope of research: this study focused on combatants who actively chose to share their stories-an act that in itself indicates certain coping resources. Therefore, there is a fundamental need for future research to examine the “avoidant” group of soldiers-those who chose not to take part in the initiative or avoided community exposure. A vital research hypothesis to be tested is whether avoidance of participation in such meetings correlates with higher levels of Post-Traumatic Stress Disorder (PTSD) or avoidance symptoms. This understanding is critical to determine whether the community model is suitable for the entire combatant population or if unique adaptations are required for soldiers who are not emotionally available for this type of exposure.

In conclusion, “Gibor BaSalon” is more than a social initiative; it is a living human laboratory for collective healing. In an era where wars become mass trauma events, the community's ability to open its doors and its heart and become an active partner in processing memory is a cornerstone of both national and personal resilience.

### 6.3 Clinical and policy implications

1. Integration in therapy: therapists treating combatants should encourage patients (at the appropriate stage) to participate in community sharing sessions as part of a treatment plan (Exposure-based intervention supplement), while processing the experience in the therapy room before and after the session.
2. Structured protocol: it is recommended to develop a facilitation protocol for “Gibor BaSalon” hosts, including tools to identify dissociation or acute distress in the speaker, and creating a “Safety Net” of available professionals.
3. Preventing moral injury: the community encounter serves as a secular “Purification Ritual.” Policymakers should encourage these spaces not only for “battle heritage,” but as a preventative mental health tool to reduce shame and guilt.

## Data Availability

The raw data supporting the conclusions of this article will be made available by the authors, without undue reservation.

## References

[1] Usbeck F. “Keep That Fan Mail Coming”: ceremonial storytelling and audience interaction in a US soldier's milblog. Z für Angl Am. (2014) 62:149–63. doi: 10.1515/zaa-2014-0018

[2] Barrios MÁB Miravalles AFi. Community-based emancipatory strategies for being resilient in chaotic times. Aula de Encuentro. (2025). doi: 10.17561/ae.v27n1.9305

[3] Ponce CA Wlodarczyk A. Community resilience and posttraumatic growth in the aftermath of collective disaster and trauma. Int J Soc Psychol. (2020) 35:229–66.

[4] Nuttman-Shwartz O. Shared resilience in a traumatic reality a new concept for trauma workers exposed personally and professionally to collective disaster. Trauma Violence Abuse. (2015) 16:466–75. doi: 10.1177/1524838014557287 25427830

[5] White M Epston D. Narrative Means to Therapeutic Ends. NewYork, NY: W. W. Norton and Company (1990).

[6] Denborough D. Moral Injury and Moral Repair: The Possibilities of Narrative Practice. Adelaide, SA: Dulwich Centre Publications (2021).

[7] Tappenden PC Shiner RL Mo F. Narrating life in the military: links between veterans' narrative processing of service experiences and their posttraumatic stress symptoms and well-being. J Trauma Stress. (2021) 35:288–301. doi: 10.1002/jts.22738 34655109

[8] Warren J. Narrative medicine. Emerg Med News. (2019) 41::10.1097/01.EEM.0000559606.48714.da. doi: 10.1097/01.EEM.0000559606.48714.da

[9] Herman JL. Trauma and Recovery. New York, NY: Basic Books (1992).

[10] Ogden P. Trauma and the body: a sensorimotor approach to psychotherapy. Br J Guid Couns. (2010) 37:514–516. doi: 10.1080/03069880903166509

[11] Jaeger J Lindblom KM Parker-Guilbert K Zoellner LA. Trauma narratives: it's what you say, not how you say it. Psychol Trauma. (2014) 6:473–81. doi: 10.1037/a0035239 25379123 PMC4217123

[12] Felman S Laub D. Testimony: Crises of Witnessing in Literature, Psychoanalysis, and History. New York, NY: Routledge (1992).

[13] LaCapra D. Writing History, Writing Trauma. Revised ed. Baltimore, MD: Johns Hopkins University Press (2014). doi: 10.56021/9781421414003

[14] Carless D. Narrative transformation among military personnel on an adventurous training and sport course. Qual Health Res. (2014) 24:1440–50. doi: 10.1177/1049732314548596 25147220

[15] Muijnck D. Narrative, memory and PTSD: A case study of autobiographical narration after trauma. Eur J Life Writ. (2022) 11:AN75–95. doi: 10.21827/ejlw.11.38659

[16] Flores RM. “Um acontecimento que ficou gravado em minha memória”: traumas, lembranças e esquecimentos nas narrativas dos pracinhas da Força Expedicionária Brasileira. Hist Guerra. (2025). doi: 10.34096/hyg.n8.14921

[17] Hutchinson J Lema JC. Ordinary and extraordinary narratives of heroism and resistance: uncovering resilience, competence and growth. Couns Psychol Rev. (2009) 24:9–20. doi: 10.53841/bpscpr.2009.24.3-4.9

[18] Frankfurt SB Russell C. Private lives, public stories, and healing from moral injury: commentary on Jones (2018) and Schaff (2018). Mil Behav Health. (2018) 6:140–2. doi: 10.1080/21635781.2018.1454369

[19] Halbwachs M. In: Coser LA, editor. On Collective Memory. Chicago, IL: University of Chicago Press (1992). doi: 10.7208/chicago/9780226774497.001.0001

[20] Krebs RR Ralston R Balzacq T Blagden D Shenhav SR. Do soldiers get a say? soldiers' views and public support for military operations in four democracies. Perspect Polit. (2025) 23:1–20. doi: 10.1017/S1537592724002214

[21] Martin TL. A Theory of Veteran Identity (thesis). University of Kentucky, Lexington, KY, United States (2017).

[22] Tomforde M. Combat soldiers and their experiences of violence: returning to post-heroic societies? In: Political Military Sociology. Berlin: Springer (2018). p. 195–214. doi: 10.1007/978-3-319-71602-2_11

[23] Morris JT. Moral Injury Among Returning Veterans: From Thank You for Your Service to a Liberative Solidarity. Lanhem, MA: Lexington Books (2021). doi: 10.5040/9781978721210

[24] Beaumont J. Commemoration in Australia: A memory orgy? Aust J Polit Sci. (2015) 50:536–44. doi: 10.1080/10361146.2015.1079939

[25] Mathers JG. Medals and American heroic military masculinity after 9/11. In:Robinson L Xu X, editor, Mediating War and Identity: Figures of Transgression in 20th and 21st Century War Representation. New York, NY: Routledge (2020). p. 37–56.

[26] Parrott SD Albright DL Eckhart N Laha-Walsh K. *U.S.* Veterans and civilians describe military news coverage as mediocre, think stories affect others more than themselves. Armed Forces Soc. (2022) 49:713–28. 10.1177/0095327X221080944

[27] Bodnar J. The Soldiers' Debate: Wars and Memory in America. Chapel Hill, NC: University of North Carolina Press (2022).

[28] Giesler MA Juarez A. “I Have Served to Tell”: a qualitative study of veterans' reactions on participating in a living library project. J Vet Stud. (2019) 5:34–44. doi: 10.21061/jvs.v5i1.148

[29] Lysychkina I Lysychkina O. Narrative as a tool for shaping military personnel's collective consciousness. Zbìrnik Naukovih Prac′. (2020).

[30] Ilagan GT. Narratives of community resilience from two villages in North Cotabato. Philipp Soc Sci Rev. (2012).

[31] Khan R Khanam A Moeen B. Exploring the role of OPT: trauma narratives and community building in times of war and crisis. Glob Soc Rev. (2024) 9:34–42. doi: 10.31703/gsr.2024(IX-II).04

[32] Shinn J. The Veteran R.E.A.D.S. Program (Resilience, Education, Advocacy, Dialogue, Service). White Paper. Columbia, MO: University of Missouri Extension (2024).

[33] Rathnayake HGMD. From sacrifice to solidarity: war veterans as catalysts for intergenerational healing through cultural revitalization. Sri Lanka J Soc Work. (2025) 9:95–105. doi: 10.4038/sljsw.v9i1.21

[34] Grimm C Terte Ide Hodgetts D Kearney S. Narratives of holistic mental health recovery in New Zealand defence force personnel. Mil Psychol. (2023) 36:650–60. doi: 10.1080/08995605.2023.2250708 37643328 PMC11622612

[35] Robinson L. Soldiers' stories of the Falklands war: recomposing trauma in memoir. Contemp Br Hist. (2011) 25:569–89. doi: 10.1080/13619462.2011.623866

[36] Braun V Clarke V. Thematic Analysis: A Practical Guide. Redwood City, CA: SAGE Publications (2021).

[37] Tzemach S. “Hero in the Living Room”: community resilience through personal stories. J Israeli Commun Stud. (2022) 15:88–104.

[38] Turner DW. Qualitative interview design: a practical guide for novice investigators *Qual*. Rep. (2010) 15:754–60. doi: 10.46743/2160-3715/2010.1178

[39] Bell E Bryman A Harley B. Business Research Methods. 6th ed. Oxford: Oxford University Press (2022). doi: 10.1093/hebz/9780198869443.001.0001

[40] Creswell JW Creswell JD. Research Design: Qualitative, Quantitative, and Mixed Methods Approaches. Redwood City, CA: SAGE Publications (2018).

[41] Creswell JW Poth CN. Qualitative Inquiry and Research Design: Choosing Among Five Approaches. Redwood City, CA: SAGE Publications (2018).

[42] Nowell LS Norris JM White DE Moules NJ. Thematic analysis: striving to meet the trustworthiness criteria. Int J Qual Methods. (2017) 16. doi: 10.1177/1609406917733847

[43] Smith J Firth J. Qualitative data analysis: the framework approach. Nurse Res. (2011) 18:52–62. doi: 10.7748/nr2011.01.18.2.52.c8284 21319484

[44] Guba EG Lincoln YS. Naturalistic Inquiry. Thousand Oaks, CA: SAGE Publications (1985). doi: 10.1016/0147-1767(85)90062-8

[45] Finn M. Local heroes: war news and the construction of ‘community' in Britain, 1914–18. Hist Res. (2008) 81:721–44.

[46] Mamon D McDonald EC Lambert J Cameron AY. Connecting veterans and their community through narrative: pilot data on a community strengthening intervention. Community Ment Health J. (2020) 56:1290–7. doi: 10.1007/s10597-019-00540-3 31907805

[47] Bion WR. Learning from Experience. Hamburg: Heinemann. (1962).

[48] Winnicott DW. The theory of the parent-infant relationship. Int J Psychoanal. (1960) 41:585–95. 13785877

[49] Jetten J Haslam C Haslam SA. The Social Cure: Identity, Health and Well-Being. Hove: Psychology Press (2012). doi: 10.4324/9780203813195

[50] Cabanes B. Le retour du soldat au XXe siècle. Rev Hist Armées. (2006) 245:4–16. doi: 10.3917/rha.245.0004

[51] Mamon D McDonald EC Lambert J Cameron AY. Using storytelling to heal trauma and bridge the cultural divide between veterans and civilians. J Loss Trauma. (2017) 22:669–80. doi: 10.1080/15325024.2017.1382653

[52] Rothberg M. The Implicated Subject: Beyond Victims and Perpetrators. Redwood City, CA: Stanford University Press (2019). doi: 10.1515/9781503609600

[53] Bruder M. Holocaustos y resiliencia. sanando heridas a través de la escritura y cuento terapéutico. Psicodebate. (2008) 8:7. doi: 10.18682/pd.v8i0.413

[54] Caplan PJ Milkiewicz H. Listening to veterans: the Welcome Johnny and Jane Home project. Psychol Serv. (2012) 9:299–306.

[55] Caruth C. Unclaimed Experience: Trauma, Narrative, and History. Baltimore, MD: Johns Hopkins University Press (1996). doi: 10.1353/book.20656

[56] Deichmann CL. Heroes for all time: connecticut civil war soldiers tell their stories. J Am History. (2018) 105:163–4. doi: 10.2307/44370313

[57] Eid J Johnsen BH Saus E-R. Trauma narratives and emotional processing. Scand J Psychol. (2005) 46:503–10. doi: 10.1111/j.1467-9450.2005.00482.x 16277651

[58] McAllister L Callaghan J Fellin LC. Masculinities and emotional expression in UK servicemen: big boys don't cry? J Gender Stud. (2019) 28:257–70. doi: 10.1080/09589236.2018.1429898

[59] Willsey K. At war with stories: a vernacular critique of the storytelling boom from American military veterans. Poet Today. (2022) 43:273–301. doi: 10.1215/03335372-9642595

[60] Paguia RRP Balongoy PAP. The ferocious fighters: understanding the lived experiences of battle-tested soldiers in combat area. Int J Soc Arts Theol. (2025) 16. doi: 10.71097/ijsat.v16.i2.6257

[61] Given LM editor. The SAGE Encyclopedia of Qualitative Research Methods. SAGE Publications (2021). doi: 10.4135/9781412963909

